# Dietary patterns associated with fall-related fracture in elderly Japanese: a population based prospective study

**DOI:** 10.1186/1471-2318-10-31

**Published:** 2010-06-01

**Authors:** Yasutake Monma, Kaijun Niu, Koh Iwasaki, Naoki Tomita, Naoki Nakaya, Atsushi Hozawa, Shinichi Kuriyama, Shin Takayama, Takashi Seki, Takashi Takeda, Nobuo Yaegashi, Satoru Ebihara, Hiroyuki Arai, Ryoichi Nagatomi, Ichiro Tsuji

**Affiliations:** 1 Graduate Medical Education Center, Tohoku University Hospital, Sendai, Japan; 2 Department of Medicine and Science in Sports and Exercise, Tohoku University Graduate School of Medicine; 3 Center for Asian Traditional Medicine, Tohoku University Graduate School of Medicine; 4 Department of Geriatrics & Gerontology Division of Brain Science, Institute of Development Aging and Cancer, Tohoku University; 5 Department of Public Health and Forensic Medicine, Tohoku University Graduate School of Medicine; 6 Department of Internal Medicine and Rehabilitation Science, Tohoku University Graduate School of Medicine

## Abstract

**Background:**

Diet is considered an important factor for bone health, but is composed of a wide variety of foods containing complex combinations of nutrients. Therefore we investigated the relationship between dietary patterns and fall-related fractures in the elderly.

**Methods:**

We designed a population-based prospective survey of 1178 elderly people in Japan in 2002. Dietary intake was assessed with a 75-item food frequency questionnaire (FFQ), from which dietary patterns were created by factor analysis from 27 food groups. The frequency of fall-related fracture was investigated based on insurance claim records from 2002 until 2006. The relationship between the incidence of fall-related fracture and modifiable factors, including dietary patterns, were examined. The Cox proportional hazards regression model was used to examine the relationships between dietary patterns and incidence of fall-related fracture with adjustment for age, gender, Body Mass Index (BMI) and energy intake.

**Results:**

Among 877 participants who agreed to a 4 year follow-up, 28 suffered from a fall-related fracture. Three dietary patterns were identified: mainly vegetable, mainly meat and mainly traditional Japanese. The moderately confirmed (see statistical methods) groups with a Meat pattern showed a reduced risk of fall-related fracture (Hazard ratio = 0.36, 95% CI = 0.13 - 0.94) after adjustment for age, gender, BMI and energy intake. The Vegetable pattern showed a significant risk increase (Hazard ratio = 2.67, 95% CI = 1.03 - 6.90) after adjustment for age, gender and BMI. The Traditional Japanese pattern had no relationship to the risk of fall-related fracture.

**Conclusions:**

The results of this study have the potential to reduce fall-related fracture risk in elderly Japanese. The results should be interpreted in light of the overall low meat intake of the Japanese population.

## Background

Fracture accidents in the elderly reduces their activity of daily life [1] and also increases mortality [2-4]. Diet is considered an important factor for the maintenance of bone health [5-7]. Many nutrients, not only calcium [8,9] and Vitamin D [10], but also phosphorus, vitamin K, strontium and magnesium [11,12], contribute to bone health Bone is a complex living tissue, however, and a wide spectrum of micronutrients also contribute to its maintenance. Moreover, diets are composed of a wide variety of foods containing complex combinations of nutrients. Therefore, surveys that examine a single nutrient in foods may not adequately account for complicated interactions and cumulative effects on human health.

Tucker *et al*. [13] and Okubo *et al*. [14] categorized diets into dietary patterns in order to clarify the relationship between diet and bone mineral density (BMD). Tucker and colleagues reported that a diet with a high fruit and vegetable content appears to have a protective effect on BMD in males while high candy consumption may be associated with low BMD. Okubo *et al*. demonstrated the possibility that a dietary pattern with high intakes of fish, fruit, and vegetables and a low intake of meats may have a beneficial effect on BMD. Tucker and Okubo's observation, however, was not extended to look for associations between dietary patterns and fractures. There is no report investigating the relation of dietary patterns and fall-related fracture. Furthermore, the population studied by Okubo *et al*. was made up of pre-menopausal women. Though Tucker *et al*. studied an elderly population, the dietary habits of people from Western versus Asian countries are entirely different. As is well known, Japanese food is characterized by rice and soy bean products, and contains many types of fish, seafood and vegetables but only small amounts of meat or dairy products [15]. Therefore, in the present study, we examined the relationship between dietary patterns and fractures in elderly Japanese living in a suburb of Sendai, one of the largest cities in Northern Japan.

## Methods

### Study population

Our study population consisted of elderly subjects living in the Tsurugaya area of Sendai, the largest city of Tohoku (North-eastern) district in Japan. At the time of the study in 2002, there were 2730 people aged over 70 years living in the area. We invited all of these people to participate in a comprehensive geriatric assessment of medical status of whom 1178 agreed to participate and provided written informed consent for a baseline assessment. Of these 1178, we excluded 213 subjects who did not agree to the follow up survey, 77 with incomplete dietary data and 11 whose cognitive level was lower than 18 in the Mini Mental State Examination (MMSE) score [16]. Therefore, 877 participants whose medical status, activities of daily living (ADL), and life style, including dietary intakes, were assessed in July 2002 were followed up for their incidence of fall related fracture until the end of July 2006. Medical doctors (specializing in rehabilitation, exercise medicine and psychiatry), pharmacists, nurses, and kinesipathists assessed their baseline characteristics.

### Assessment of dietary intake

The short version of a previously published self-administered food frequency questionnaire (FFQ) [17] was used for the present study. This included 75 food items with specified serving sizes that were described by natural portions or standard weight and volume measures of the servings commonly consumed in our study population. For each food item, participants indicated their mean frequency of consumption over the past year in terms of the specified serving size by checking 1 of the 7 frequency categories ranging from "almost never" to "2 or more times/d". Frequency data was converted to the gram intake as described previously [18]. The mean daily intake of nutrients was calculated using an ad hoc computer program developed to analyze the questionnaire. In the validity study of the present FFQ, the questionnaire provided close estimation of nutrients compared to the 3-day diet record [19].

### Assessment of other variables

In addition to diet, we investigated the following factors related to fractures according to a WHO report [20]: age, gender, BMI calculated as weight (kg)/height (m) squared, MMSE as a measure of cognitive function, the medical outcome study questionnaire (MOS) [21] for ADL, smoking, past falls, past history of apoplexy, diabetes mellitus, osteoporosis, renal disease and cancer. Also we investigated the use of stabilizers, hypnotics, steroids and hormone replacement therapy (HRT). Anthropometric measurements i.e. height and body weight were recorded using a standard protocol. Alcohol consumption and use of supplements including calcium and multivitamins were assessed from the FFQ.

### Diagnosis of fracture

The incidence and causes of any fractures were investigated based on insurance claim records from July 2002 until July 2006. Fracture data was available on all 877 participants including 39 subjects who had died in the follow-up period. All clinical records of patients with fractures were reviewed by a physician (R.N.). Cases involving traumatic fracture such as traffic accidents were included in the "Non fall-related fracture group".

### Statistical analysis

Factor analysis was used to derive dietary patterns and to determine factor loadings for each of the 27 food subgroups. Factor analysis is a statistical method used to describe variability among observed variables in terms of fewer unobserved variables called factors [22]. Factors were rotated with varimax rotation to maintain uncorrelated factors and enhance interpretability. Dietary patterns were named according to the nature of the food groups loading highest for each of the factors. For each pattern and each participant, we calculated a factor score by summing the consumption of each food item weighted by its factor loading [18]. The subjects were divided into tertiles according to the factor score as follows: unconfirmed (the first tertile: T1), moderately confirmed (the second tertile: T2) and confirmed (the third tertile: T3) according to the factor score of each dietary pattern.

A simple logistical regression model was used to examine the relationships between the risk of fall-related fracture and general characteristics. Sample characteristics for T1, T2 and T3 in each dietary pattern were statistically analyzed using the parametric test. The Cox proportional hazards regression model was also used to examine the relationships between other variables mentioned above and the incidence of fall-related fractures with adjustment for age, gender [23], BMI [24] and energy intake. Hazard ratio (HR) and 95% CIs were calculated. The probabilities of being fracture free were estimated using the Kaplan-Meier product-limit method. Fracture free numbers were calculated from the date of enrolment to the date of fracture onset, or cut-off date for participants alive at the time of closure of the dataset. A significant difference was defined as p < 0.05. All statistical analyses were performed using the Statistical Analysis System 9.1 edition for WINDOWS (SAS Institute Inc, Cary, NC)

### Ethics

The Institutional Review Board of Tohoku University Graduate School of Medicine approved the protocol of the study. Written informed consent was obtained from study participants. The study was not registered to any clinical trial registration websites because the study started in 2001 and the recruitment of participants was completed in 2002.

## Results

### Study population

**Figure 1:**
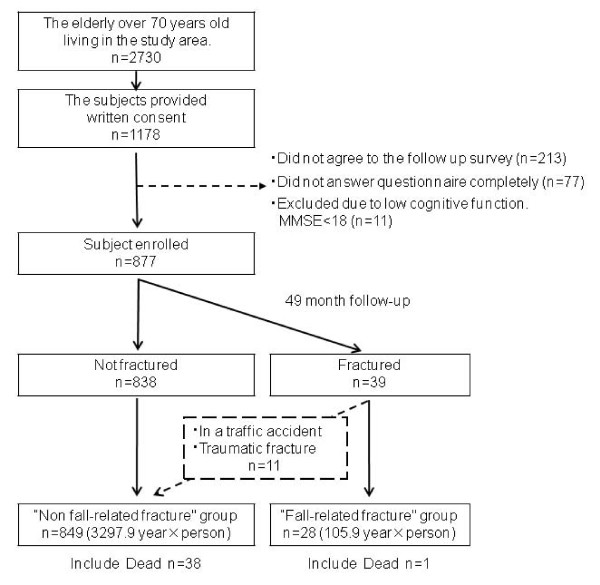
**Study protocol**. Of these 1178, we excluded 213 subjects who did not agree to the follow up survey, 77 with incomplete dietary data and 11 whose cognitive level was lower than 18 in the Mini Mental State Examination (MMSE) score. Therefore, 877 participants whose medical status, activities of daily living (ADL), and life style including dietary intakes were assessed in July 2002 were followed up until the end of July 2006.

Of the 877 registered participants, 39 had suffered a fracture by the end of July 2006. Eleven participants had fractures due to traffic accident or other injuries. Therefore, we compared the remaining 28, who fractured due to a fall, to the other 849 participants who did not have a fall related fracture within our follow up period (Figure 1). Eleven persons who had fractures due to traffic accident or other injuries were included in the non fall-related fracture group. Their background, including age, height, weight, BMI, MMSE, MOS, energy intake, gender, history of stroke, diabetes, kidney disease, osteoporosis, cancer, use of tranquilizers, sleeping pills, steroids, supplements, HRT, smoking habit or alcohol, and falls in the previous 6 months were compared between the fall-related fracture and non fall-related fracture group. There were statistically significant differences in age (a mean of 82.3 years old in the fracture group and 79.1 years old in the non fall-related fracture group, p = 0.01) and smoking habit (a rate of 21.4% in the fall-related fracture group and 43.7% in the non fall-related fracture group, p = 0.026) (Table 1).

**Table 1:** General characteristics between the fracture and non fracture groups.

	Fall-related fracture group (n = 28)			Non fall-related fracture group (n = 849)			p value
Age (years old)	82.3	±	5.9	79.1	±	4.6	**0.001**
Height (cm)	151.9	±	9.3	154.6	±	8.7	0.121
Weight (kg)	54.2	±	9.0	57.0	±	9.6	0.121
BMI (kg/m^2^)	23.4	±	3.1	23.8	±	3.3	0.528
MMSE	26.6	±	2.7	27.5	±	2.3	0.058
MOS score	3.9	±	1.8	4.3	±	1.7	0.227
Energy intake	2025	±	640	1998	±	495	0.782
Gender (male)		28.6%			44.8%		0.096
Stroke history		7.1%			5.3%		0.671
Diabetes history		17.9%			13.8%		0.541
Kidney disease history		0.0%			7.3%		0.997
Osteoporosis history		3.6%			7.3%		0.462
Cancer history		21.4%			12.8%		0.192
Use of stabilizer		10.7%			13.3%		0.691
Use of hypnotic		7.1%			10.7%		0.549
Use of steroid		3.6%			4.0%		0.908
Use of supplement		10.7%			18.1%		0.589
Use of HRT		39.3%			3.7%		0.998
Smoking habit		21.4%			43.7%		**0.026**
Drinking habit		35.7%			52.1%		0.085
Experience of falls in previous 6 months		25.0%			16.5%		0.252

Variable are presented as mean ± SD or %.

BMI; body mass index, MMSE; Mini Mental State Examination, MOS score; medical outcome study questionnaire, HRT; Hormone Replacement Therapy.

Analysis by Simple logistic regression model

Data in bold are p < 0.05

### Dietary patterns identified

The factor-loading matrices are shown in Table 2. Factor 1 is loaded on a high consumption of vegetables, seaweeds, mushrooms, soy products and salt. Therefore, factor 1 was designated the Vegetable pattern. Factor 2 was designated the Meat pattern because it was loaded with a high consumption of meat (chicken, pork and beef), processed meat (ham, sausage, liver paste) and seafood (squid, octopus, shrimp, lobster and shellfish). Factor 3 was heavily loaded with rice and Miso soup intake. Also, this factor was mildly loaded with Natto (fermented soybean, a typical traditional soy product in East Asia). Therefore, we designated this factor as the Traditional Japanese pattern. The scree plots dropped on 2.5 after the third factor, factor 1 (eigenvalue 5.0) explaining 15.5% of the variability, factor 2 (3.0) explaining 7.3%, and factor 3 (2.8) explaining 7.2%.

**Table 2:** Factor analysis for patterns identified (Factor-loading matrix).

	Factor 1: The Vegetable pattern	Factor 2: The Meat pattern	Factor 3: The Traditional Japanese pattern
Radish, Turnip	**0.72**	0.12	0.08
Carrot, Pumpkin	**0.71**	0.01	0.00
Vegetables with light green leaves	**0.64**	0.25	-0.10
Salt intake	**0.59**	0.16	0.11
Vegetable with green leaves	**0.56**	0.19	-0.06
Seaweed	**0.53**	0.15	0.04
Potato	**0.52**	0.00	0.22
Mushroom	**0.51**	0.17	-0.12
Soy product	**0.51**	0.03	0.34
Tomato	**0.49**	0.01	-0.03
Fish	**0.36**	0.26	0.05
Egg	**0.21**	0.20	0.13
Pork, beef, ham, liver	0.08	**0.68**	0.09
Chicken	0.04	**0.61**	0.19
Shellfish, Cuttlefish, Octopus, Shrimp	0.13	**0.53**	-0.05
Noodle	0.22	**0.43**	-0.12
Coffee	0.11	**0.33**	-0.01
Coke	-0.09	**0.31**	0.12
Milk	0.22	**-0.25**	0.15
Pickled vegetable	0.22	**0.24**	0.09
Black tea, Oolong tea	0.08	**0.14**	-0.08
Miso soup	0.17	0.07	**0.72**
Rice	-0.04	0.12	**0.69**
Natto (fermented soybeans)	0.37	-0.05	**0.43**
Persimmon, Strawberry, Kiwi	0.29	-0.01	**-0.41**
Citrus	0.35	-0.07	**-0.39**
Green tea	0.13	-0.03	**0.31**

Percentage of variance (%)	15.5%	7.3%	7.2%

Data for 877 subjects from the FFQ

Data highlighted in bold

Sample characteristics for T1, T2 and T3 in each dietary pattern are displayed in Additional file 1, Table S3. ADL (MOS), past history of diabetes and use of supplements showed a significant trend in the Vegetable pattern. Age, height, weight, ADL, % of male, past history of osteoporosis (decreased) and cancers (decreased), smoking and drinking habits showed significant trends in the Meat pattern. Finally, Height, weight, ADL (MOS), percentage of male, past history of stroke, diabetes, osteoporosis (decreased) and cancers (decreased), smoking and drinking habits showed significant trends in the Traditional Japanese pattern.

Intake of energy, total, animal and vegetable proteins, Vitamins Bs, C, D, K and electrolytes showed significant increase from T1 to T3 in all three dietary patterns. Alcohol intake also significantly increased from T1 to T3 in the Meat pattern and the Traditional Japanese pattern (Additional file 2, Table S4).

### Hazard ratio of fall related fractures

**Figure 2:**
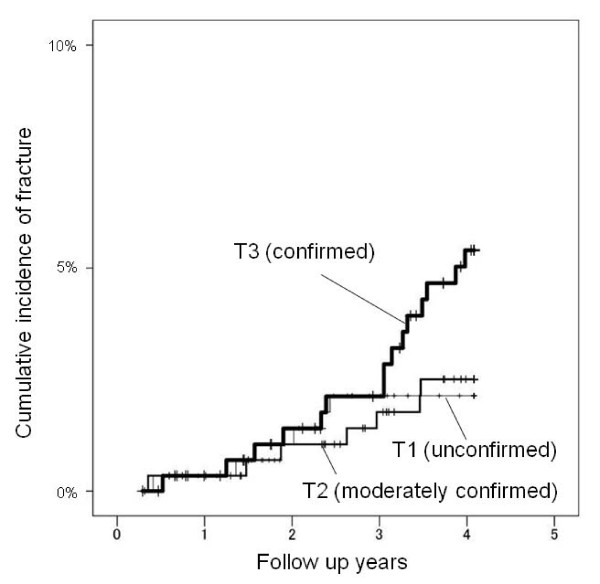
**The accumulated rate of fall-related fracture onset in each tertile of the Vegetable pattern**. The cumulative fall-related fracture incidence in T3 (confirmed) group of the Vegetable pattern is visibly higher than T1 or T2.

**Figure 3:**
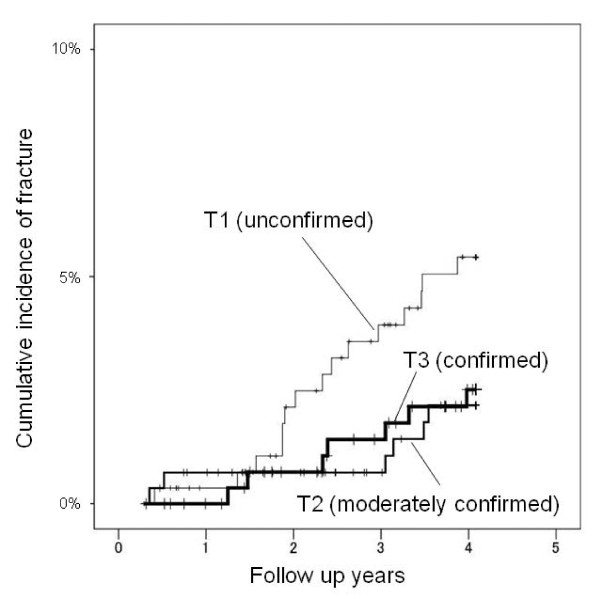
**The accumulated rate of fall-related fracture onset in each tertile of the Meat pattern**. The cumulative fall-related fracture incidence in T1 (unconfirmed) group of the Meat pattern is higher than T2 or T3.

The hazard ratios (HR) of fall-related fractures in each dietary pattern are shown in Table 3. The vegetable pattern showed a significant trend for the risk of fall-related fracture. In this pattern, the HR of T3 (confirmed group) compared to T1 (unconfirmed group) was 2.67 (95% CI 1.03 - 6.90) when data was adjusted for age, gender and BMI. The p trend in the Meat pattern for fall-related fracture risk was 0.056 when age, gender, BMI and energy intake were adjusted. The HR of T2 versus T1 in the Meat pattern was 0.36 (95% CI 0.13 - 0.94). Figure 2 indicates the accumulated rate of fall-related fracture onset in tertiles of the Vegetable pattern. The cumulative fall-related fracture incidence in T3 (confirmed) of the Vegetable pattern was higher than T1 or T2. Figure 3 shows that the cumulative fracture incidence in T1 (unconfirmed) of the Meat pattern is higher than T2 or T3. Finally, there was no significant tendency towards fall-related fracture risk in the Traditional Japanese pattern.

**Table 3:** Hazard ratio (95%CI) of fall-related fracture in each dietary pattern.

				(3403.8 year*person)
	**T1** **(unconfirmed)**	**T2** **(moderately confirmed)**	**T3** **(confirmed)**	***p*** **for trend**

The Vegetable pattern				
Model 1 Hazard Ratio	1.00 (Reference)	1.13 (0.38-3.36)	**2.67 (1.03-6.90)**	**0.025**
Model 2 Hazard Ratio	1.00 (Reference)	1.11 (0.37-3.31)	**2.66 (1.03-6.87)**	**0.025**
Model 3 Hazard Ratio	1.00 (Reference)	1.12 (0.37-3.39)	2.64 (0.93-7.47)	**0.044**
Model 4 Hazard Ratio	1.00 (Reference)	1.10 (0.36-3.34)	2.62 (0.93-7.41)	**0.044**
The Meat pattern				
Model 1 Hazard Ratio	1.00 (Reference)	0.43 (0.17-1.10)	0.58 (0.23-1.47)	0.211
Model 2 Hazard Ratio	1.00 (Reference)	0.43 (0.17-1.12)	0.58 (0.23-1.47)	0.212
Model 3 Hazard Ratio	1.00 (Reference)	**0.36 (0.13-0.94)**	0.36 (0.12-1.06)	0.056
Model 4 Hazard Ratio	1.00 (Reference)	**0.36 (0.14-0.96)**	0.36 (0.12-1.06)	0.057
The Traditional Japanese pattern				
Model 1 Hazard Ratio	1.00 (Reference)	0.79 (0.33-1.91)	0.80 (0.28-2.28)	0.646
Model 2 Hazard Ratio	1.00 (Reference)	0.81 (0.33-1.96)	0.81 (0.29-2.30)	0.661
Model 3 Hazard Ratio	1.00 (Reference)	0.75 (0.31-1.81)	0.75 (0.26-2.17)	0.561
Model 4 Hazard Ratio	1.00 (Reference)	0.77 (0.32-1.86)	0.76 (0.26-2.19)	0.579

Analysis by Cox proportional hazards model

Model 1: Adjusted by age, gender and BMI

Model 2: Adjusted by Model 1 variable and experience of falls in previous 6 month

Model 3: Adjusted by age, gender, BMI and Energy intake

Model 4: Adjusted by Model 3 variable and experience of falls in previous 6 month

Data in bold are **p < 0.05**

Among the 75 food items, vegetables with light green leaves such as lettuce and cabbage (HR = 0.97 for 1 g intake, 95% CI 0.94 - 1.00, p = 0.023) were found to significantly reduce the risk of fall-related fracture when adjusted for age, gender, BMI and energy intake (Table 4). In contrast, seaweed (HR = 1.04, 95% CI 1.00 - 1.08), root vegetables (HR = 1.02, 95% CI 1.00 - 1.03), ice cream (HR = 1.01, 95% CI 1.00 - 1.01) and snacks (HR = 1.04, 95% CI 1.01 - 1.07, p = 0.008) significantly increased the risk of fall-related fracture. The significance of seaweed and roots vegetables was removed when data was adjusted for energy intake. No other food, including dairy products, shellfish, fish, fruit, soybeans and meat showed any relation to the risk of fall-related fracture (p values were over 0.05).

**Table 4:** Hazard ratio(95%CI) of fall related fracture for each food item (g/day).

			(3403.8 year*person)
	**Hazard ratio (95%CI)**		**p value**

Seaweed			
Model 1 Hazard Ratio	**1.04**	**(1.00 - 1.08)**	**0.031**
Model 2 Hazard Ratio	**1.04**	**(1.00 - 1.08)**	**0.032**
Model 3 Hazard Ratio	1.04	(1.00 - 1.08)	0.054
Model 4 Hazard Ratio	1.04	(1.00 - 1.08)	0.056
Root vegetables			
Model 1 Hazard Ratio	**1.02**	**(1.00 - 1.03)**	**0.043**
Model 2 Hazard Ratio	**1.02**	**(1.00 - 1.03)**	**0.041**
Model 3 Hazard Ratio	1.01	(1.00 - 1.03)	0.072
Model 4 Hazard Ratio	1.01	(1.00 - 1.03)	0.069
Snacks, Rice cake, Okonomiyaki			
Model 1 Hazard Ratio	**1.04**	**(1.01 - 1.07)**	**0.008**
Model 2 Hazard Ratio	**1.04**	**(1.01 - 1.07)**	**0.008**
Model 3 Hazard Ratio	**1.04**	**(1.01 - 1.07)**	**0.014**
Model 4 Hazard Ratio	**1.04**	**(1.01 - 1.07)**	**0.016**
Ice cream			
Model 1 Hazard Ratio	**1.01**	**(1.00 - 1.01)**	**0.003**
Model 2 Hazard Ratio	**1.01**	**(1.00 - 1.01)**	**0.004**
Model 3 Hazard Ratio	**1.01**	**(1.00 - 1.01)**	**0.006**
Model 4 Hazard Ratio	**1.01**	**(1.00 - 1.01)**	**0.008**
Vegetables with light green leaves			
Model 1 Hazard Ratio	**0.97**	**(0.94 - 1.00)**	**0.045**
Model 2 Hazard Ratio	**0.97**	**(0.94 - 1.00)**	**0.047**
Model 3 Hazard Ratio	**0.97**	**(0.94 - 1.00)**	**0.023**
Model 4 Hazard Ratio	**0.97**	**(0.94 - 1.00)**	**0.025**

Analysis by Cox proportional hazards model

Model 1: Adjusted by age, gender and BMI

Model 2: Adjusted by Model 1 variable and experience of falls in previous 6 month

Model 3: Adjusted by age, gender, BMI and Energy intake

Model 4: Adjusted by Model 3 variable and experience of falls in previous 6 month

Data in bold are **p < 0.05**

Non significant foods were excluded from the table for simplicity.

## Discussion

The present study is a population based prospective study investigating the relationship between dietary patterns and fall-related fractures in elderly Japanese. Three dietary patterns that appeared in our study are similar to the study of Shimazu et al. who studied Japanese middle age to old age (from 40 to 79) [25]. The Vegetable pattern showed a significant trend for the risk of fall-related fracture. The T3 (confirmed group) showed a significant increase in fall-related fracture risk compared to T1 (unconfirmed group) in the Vegetable pattern. In analysis of each food item, vegetables with light green leaves reduced the fall related fracture risk whereas root vegetables and seaweeds increased the risk. Therefore, not all vegetables increases the risk of fall-related fracture, though the Vegetable pattern showed a significant risk increase overall.

In contrast, T2 (moderately confirmed group) in the Meat pattern showed a significant decrease in fall-related fracture risk compared to T1 (unconfirmed group). The trend shown in the meat pattern can be interpreted as T1 group has a tendency to increased risk of fall-related fracture relative to T2 or T3 (see Figure 3).

Our results in dietary pattern analysis appear to contradict previous reports investigating the relationship between dietary patterns and BMD. Tucker *et al*. [13] reported that a dietary pattern with a high consumption of fruit, vegetables and cereals resulted in greater BMD, while Okubo *et al*. [14] showed that a Western pattern with a high intake of fat, meat, butter and seasonings was negatively associated with BMD. Single food item analysis in our study also showed that the variety of vegetables reduces the risk of fall related fracture. Only Xu *et al*. [26] reported that a high intake of meat at a young age reduced the risk of forearm fracture in postmenopausal women. No other researcher has indicated a relationship between the intake of meat and bone health.

Discrepancies between the present and previous studies may be partially explained by differences in population characteristics. All participants in our study were Japanese older than 70 years. The mean meat intake in Japan was only 77.5 g/day in 2002 [27] whereas it reached 242 g/day in the USA in 2000 [28]. Our results should be interpreted as data from a population with low meat consumption. Some nutrients such as proteins and Vitamin Bs contained in meats are known as protective factors for fracture. Figure 2 suggests that the cumulative fall-related fracture incidence in T3 (confirmed) of the Vegetable pattern was higher than T1 or T2. In addition, Figure 3 suggests that the cumulative fracture incidence in T1 (unconfirmed) of the Meat pattern is higher than T2 or T3. In other words, excessive reliance on low meat or high vegetable intake may cause nutritional imbalances and increase the fracture risk in such a population. Nutritional analysis showed an increasing intake of energy, proteins, Vitamin Bs, C, D, K, electrolytes, folic acid and salt from T1 (unconfirmed) to T3 (confirmed) in any dietary pattern. Therefore, all these nutritional factors, not particular ones, may synergistically contribute to the difference of fracture risk among the three dietary patterns.

Dietary patterns should be interpreted in light of regional background. Japanese food culture has been affected by surrounding Asian countries over many years. Interestingly, the greatest naturalist in Chinese history Li Zizhen (1518 to 1593 AD), reported that animal meat such as beef, ram and quail would strengthen bone and muscles in his famous textbook "the General Catalogue of Herbs [29]". Moreover, he stated that light green leaves such as lettuce and cabbage were beneficial for bone health. Our results in a population-based prospective investigation using multivariate analysis may agree more with Li Zizhen than other recent studies.

Our study has several limitations. The number of participants included in the statistical analysis was 877, and the number of fall-related fractures was only 28. Therefore, we were able to adjust few factors in our analysis though many more factors are known to influence the risk of fracture. Also, the limited sample size may affect the statistical detection power. Secondly, though the study design was prospective, dietary data depended on a single cross-sectional investigation in 2002. At that time, all the participants were 70 years old or more, and they were followed for only four years. Therefore, the present study does not reflect long-term dietary habits. Diet at a young age may more strongly influence bone health [26,30]. However, it is very difficult to avoid biases in longitudinal investigations of lifestyle including diet over decades. The FFQ we used was validated against a 3-day diet record in women, designed to give an accurate description of short-term intake information rather than long-term dietary habits.

Though prior falls were known to lead to increasing risk of subsequent falls [31,32], we did not record the number of these falls. The Hazard Ratio of fracture, however, showed no difference after adjustment for experience of falls in the previous 6 months. Though our study was conducted on a particular cohort with certain characteristics (people living in a specific region with official health-care insurance), almost all subjects in Japan use the official medical insurance service we consulted. Therefore, our population is representative of the elderly throughout Japan. This is different to other countries i.e. USA. Finally, age at menopause is known to influence BMD [33] but it was not investigated in the present study.

Despite these limitations, the present study suggests that the impact of dietary patterns in the elderly should not be neglected when assessing the risk of fracture. In a population with low meat consumption, such as elderly Japanese, the moderate consumption of meats may reduce the risk of fall-related fracture. In discussions of diet and health, regional dietary habits should be taken into account.

## Conclusion

Dietary patterns were related to the risk of fracture in elderly Japanese. The Vegetable pattern increased the risk of fracture. The Meat pattern had a tendency to reduce the risk of fall-related fracture. These results should be interpreted in light of overall low meat consumption in Japan.

## Competing interests

The authors declare that they have no competing interests.

## Authors' contributions

YM, KN, KI and NT were responsible for analysis and interpretation of data, and preparation of the manuscript. The first two authors, YM and KN contributed equally to the study. KN and KI were also responsible for the study concept and design. NT carried out the statistical analysis. SK, NY, HA, RN and IT were responsible for the study design. NN and AH are clinical investigators and they contributed to the data analysis. ST, TS and TT contributed to the preparation of the manuscript. All authors read and approved the final version of the manuscript.

## Pre-publication history

The pre-publication history for this paper can be accessed here:

http://www.biomedcentral.com/1471-2318/10/31/prepub

## Supplementary Material

Additional file 1 **Table S3: Characteristics of subjects in each tertile of identified dietary patterns**. Click here for file

Additional file 2 **Table S4: Nutrition intake of subjects in each tertile of identified dietary patterns**. Click here for file
