# Supplementary material for: Dietary patterns associated with fall-related fracture in elderly Japanese: a population based prospective study
Source: BMC Geriatr. 2010 Jun 1;10:31. doi: 10.1186/1471-2318-10-31 (PMC2895588; doi:10.1186/1471-2318-10-31)
Supplement: Additional file 1 — Table S3: Characteristics of subjects in each tertile of identified dietary patterns. [file 1471-2318-10-31-S1.PDF]

Additional file 1

Table S3—Characteristics of subjects in each tertile of identified dietary patterns

	The Vegetable pattern				The Meat pattern				The Traditional Japanese pattern			
	T1 (unconfirmed)	T2 (moderately confirmed)	T3 (confirmed)	*p* for trend	T1 (unconfirmed)	T2 (moderately confirmed)	T3 (confirmed)	*p* for trend	T1 (unconfirmed)	T2 (moderately confirmed)	T3 (confirmed)	*p* for trend
Number of subjects	292	292	293	—	292	292	293	—	292	292	293	—
Factor score	-2.51〜-0.47	-0.47〜0.33	0.34〜5.70	—	-2.61〜-0.51	-0.51〜0.23	0.23〜4.98	—	-2.54〜-0.43	-0.43〜0.38	0.38〜3.88	—
Age (years old)	76.2 ± 4.7	76.2 ± 4.8	75.7 ± 4.6	0.27	76.6 ± 4.8	75.8 ± 4.4	75.6 ± 4.8	**<0.01**	76.0 ± 4.5	76.3 ± 4.9	75.8 ± 4.7	0.56
Height (cm)	154.4 ± 8.7	153.9 ± 8.9	155.1 ± 8.7	0.34	152.4 ± 8.7	153.7 ± 8.1	157.3 ± 8.7	**<0.001**	151.0 ± 7.7	154.2 ± 9.0	158.3 ± 8.0	**<0.001**
Weight (kg)	56.8 ± 9.7	56.4 ± 9.4	57.6 ± 9.7	0.37	55.6 ± 9.3	56.4 ± 9.5	58.8 ± 9.7	**<0.001**	54.9 ± 9.1	56.5 ± 9.8	59.4 ± 9.4	**<0.001**
BMI (kg/m2)	23.8 ± 3.5	23.7 ± 3.1	23.9 ± 3.3	0.82	23.9 ± 3.4	23.8 ± 3.3	23.7 ± 3.2	0.41	24.1 ± 3.6	23.7 ± 3.4	23.7 ± 3.0	0.11
MMSE	27.2 ± 2.5	27.6 ± 2.1	27.5 ± 2.2	0.11	27.2 ± 2.4	27.7 ± 2.1	27.5 ± 2.3	0.08	27.4 ± 2.3	27.4 ± 2.1	27.5 ± 2.4	0.79
MOS score	4.0 ± 1.9	4.3 ± 1.6	4.5 ± 1.7	**<0.01**	4.0 ± 1.7	4.3 ± 1.7	4.6 ± 1.7	**<0.001**	4.0 ± 1.9	4.2 ± 1.7	4.6 ± 1.5	**<0.001**
Gender (male)	43.2%	40.8%	48.8%	0.15	32.2%	35.6%	64.9%	**<0.001**	18.2%	40.8%	73.7%	**<0.001**
Stroke history	6.5%	3.4%	6.1%	0.93	5.5%	4.8%	5.8%	0.82	2.4%	6.9%	6.8%	**0.02**
Diabetes history	11.6%	12.0%	18.1%	**0.02**	15.1%	12.0%	14.7%	0.99	14.0%	12.3%	15.4%	0.63
Kidney disease history	6.5%	5.5%	9.2%	0.18	8.2%	6.5%	6.5%	0.44	8.2%	8.2%	4.8%	0.10
Osteoporosis history	17.8%	18.2%	19.8%	0.53	23.6%	17.5%	14.7%	**<0.01**	24.3%	19.9%	11.6%	**<0.001**
Cancer history	17.5%	12.7%	15.7%	0.61	18.2%	17.1%	10.6%	**<0.01**	18.5%	18.2%	9.2%	**<0.01**
Use of stabilizer	13.7%	11.6%	14.3%	0.78	16.4%	11.3%	12.0%	0.14	15.1%	12.3%	12.3%	0.33
Use of hypnotic	11.6%	10.6%	9.6%	0.41	9.9%	12.7%	9.2%	0.67	12.7%	9.9%	9.2%	0.18
Use of steroid	4.5%	4.1%	3.4%	0.52	3.1%	3.4%	5.5%	0.13	3.4%	4.1%	4.4%	0.53
Use of supplement	12.3%	20.2%	21.2%	**<0.01**	18.8%	18.8%	16.0%	0.35	16.1%	17.8%	19.8%	0.24
Use of HRT	8.9%	9.5%	3.9%	0.12	9.1%	7.1%	5.5%	0.29	8.3%	6.1%	8.8%	0.92
Smoking habit	47.3%	40.1%	41.6%	0.19	33.6%	37.3%	58.0%	**<0.001**	26.4%	40.4%	62.1%	**<0.001**
Drinking habit	50.7%	52.4%	51.5%	0.85	40.1%	49.3%	65.2%	**<0.001**	41.1%	50.0%	63.5%	**<0.001**
Experience of falls in previous 6 month	18.1%	18.1%	14.7%	0.25	18.3%	17.0%	15.5%	0.36	20.8%	14.9%	15.1%	0.08

Variable are presented as mean ± SD or %.

BMI; body mass index, MMSE; Mini Mental State Examination, MOS score; medical outcome study questionnaire, HRT; Hormone Replacement Therapy.

Analysis by ANOVA or Square test

Data in bold are p<0.05
