# Supplementary material for: Dietary patterns associated with fall-related fracture in elderly Japanese: a population based prospective study
Source: BMC Geriatr. 2010 Jun 1;10:31. doi: 10.1186/1471-2318-10-31 (PMC2895588; doi:10.1186/1471-2318-10-31)
Supplement: Additional file 2 — Table S4: Nutrition intake of subjects in each tertile of identified dietary patterns. [file 1471-2318-10-31-S2.PDF]

Additional file 2

Table 4—Nutrition intake of subjects in each tertile of identified dietary patterns

	The Vegetable pattern				The Meat pattern				The Traditional Japanese pattern			
	T1 (unconfirmed)	T2 (moderately confirmed)	T3 (confirmed)	*p* for trend	T1 (unconfirmed)	T2 (moderately confirmed)	T3 (confirmed)	*p* for trend	T1 (unconfirmed)	T2 (moderately confirmed)	T3 (confirmed)	*p* for trend
Energy intake (kcal)	1777 ± 479	1982 ± 461	2238 ± 450	**<0.001**	1727 ± 393	1932 ± 428	2337 ± 470	**<0.001**	1788 ± 471	1983 ± 437	2225 ± 492	**<0.001**
Total Protein (g)	69.6 ± 20.1	81.4 ± 19.7	95.1 ± 21.2	**<0.001**	68.4 ± 17.8	79.2 ± 17.7	98.5 ± 21.7	**<0.001**	75.4 ± 21.9	81.6 ± 20.5	89.2 ± 23.9	**<0.001**
Animal protein (g)	40.8 ± 16.3	47.9 ± 16.7	56.1 ± 17.9	**<0.001**	37.6 ± 13.9	46.4 ± 13.8	60.7 ± 18.1	**<0.001**	45.4 ± 16.5	48.0 ± 16.8	51.5 ± 20.2	**<0.001**
Vegetable protein (g)	28.8 ± 7.2	33.5 ± 6.5	39.0 ± 7.7	**<0.001**	30.8 ± 7.1	32.8 ± 7.7	37.8 ± 8.3	**<0.001**	30.0 ± 8.1	33.7 ± 7.4	37.7 ± 7.3	**<0.001**
Vitamin B1 (mg)	0.7 ± 0.2	0.9 ± 0.2	1.1 ± 0.2	**<0.001**	0.8 ± 0.2	0.9 ± 0.2	1.1 ± 0.2	**<0.001**	0.9 ± 0.2	0.9 ± 0.2	1.0 ± 0.2	**<0.001**
Vitamin B2 (mg)	1.3 ± 0.4	1.5 ± 0.4	1.8 ± 0.4	**<0.001**	1.4 ± 0.4	1.5 ± 0.4	1.7 ± 0.4	**<0.001**	1.4 ± 0.4	1.5 ± 0.4	1.7 ± 0.4	**<0.001**
Vitamin B6 (mg)	1.2 ± 0.4	1.4 ± 0.3	1.8 ± 0.4	**<0.001**	1.2 ± 0.4	1.4 ± 0.4	1.7 ± 0.4	**<0.001**	1.3 ± 0.4	1.4 ± 0.4	1.6 ± 0.5	**<0.001**
Vitamin B12 (mg)	9.7 ± 5.3	11.8 ± 5.9	14.0 ± 6.4	**<0.001**	9.0 ± 4.7	11.3 ± 4.9	15.2 ± 6.9	**<0.001**	11.1 ± 5.9	11.6 ± 5.6	12.8 ± 6.8	**<0.01**
Vitamin C (mg)	95.1 ± 28.4	129.9 ± 27.3	169.5 ± 37.6	**<0.001**	121.0 ± 43.6	131.3 ± 42.3	142.3 ± 42.7	**<0.001**	139.1 ± 49.0	125.0 ± 41.0	130.5 ± 39.6	**0.02**
Vitamin D (μg)	14.0 ± 8.0	17.2 ± 8.7	21.7 ± 10.8	**<0.001**	14.8 ± 9.1	17.5 ± 9.5	20.5 ± 9.8	**<0.001**	16.8 ± 9.4	17.4 ± 8.7	18.6 ± 11.0	**0.03**
Vitamin K (μg)	380.3 ± 134.0	491.1 ± 132.8	606.5 ± 150.4	**<0.001**	459.3 ± 162.7	492.4 ± 164.7	526.5 ± 167.5	**<0.001**	424.2 ± 151.8	486.6 ± 148.8	567.2 ± 168.6	**<0.001**
Calcium (mg)	520.0 ± 160.2	647.1 ± 167.8	782.1 ± 169.7	**<0.001**	621.4 ± 199.9	650.2 ± 197.4	677.9 ± 191.2	**<0.001**	615.5 ± 197.7	638.1 ± 181.2	695.9 ± 204.3	**<0.001**
Sodium (mg)	4098 ± 1095	5069 ± 1254	6082 ± 1479	**<0.001**	4334 ± 1235	4977 ± 1327	5939 ± 1529	**<0.001**	4719 ± 1520	5070 ± 1483	5463 ± 1466	**<0.001**
Magnesium (mg)	237.7 ± 59.2	293.7 ± 55.4	356.8 ± 63.0	**<0.001**	259.7 ± 67.0	289.6 ± 68.1	338.9 ± 73.0	**<0.001**	273.5 ± 79.4	291.1 ± 67.3	323.8 ± 74.3	**<0.001**
Phosphorus (mg)	1051 ± 285	1253 ± 287	1484 ± 311	**<0.001**	1090 ± 292	1230 ± 292	1467 ± 333	**<0.001**	1172 ± 339	1246 ± 304	1370 ± 355	**<0.001**
Folic acid (μg)	282.2 ± 84.9	364.4 ± 87.6	461.9 ± 111.6	**<0.001**	325.3 ± 108.3	363.7 ± 101.7	419.6 ± 130.4	**<0.001**	355.4 ± 124.6	357.6 ± 112.3	395.7 ± 120.2	**<0.001**
Salt intake (g)	10.3 ± 2.8	12.8 ± 3.2	15.3 ± 3.7	**<0.001**	10.9 ± 3.1	12.5 ± 3.4	15.0 ± 3.9	**<0.001**	11.9 ± 3.8	12.8 ± 3.7	13.7 ± 3.7	**<0.001**
Alcohol intake (g)	9.2 ± 20.1	8.4 ± 18.8	7.8 ± 19.1	0.39	4.7 ± 15.1	7.8 ± 19.2	12.8 ± 22.2	**<0.001**	4.9 ± 14.2	8.5 ± 18.9	12.0 ± 23.1	**<0.001**

Variable are presented as mean ± SD

Analysis by ANOVA

Data in bold are p<0.05
